# Molecular characterization of an unusual new plant RNA virus reveals an evolutionary link between two different virus families

**DOI:** 10.1371/journal.pone.0206382

**Published:** 2018-10-22

**Authors:** Sun-Jung Kwon, Gug-Seoun Choi, Boram Choi, Jang-Kyun Seo

**Affiliations:** 1 Horticultural and Herbal Crop Environment Division, National Institute of Horticultural and Herbal Science, Wanju, Republic of Korea; 2 Department of International Agricultural Technology and Institutes of Green Bio Science and Technology, Seoul National University, Pyeongchang, Republic of Korea; University of California, Riverside, UNITED STATES

## Abstract

An unusual novel plant virus provisionally named goji berry chlorosis virus (GBCV) was isolated from goji berry plants (*Lycium chinense* Miller) showing chlorosis symptoms and its complete genome sequence was determined. The viral genome consists of a positive-sense single-stranded RNA of 10,100 ribonucleotides and contains six open reading frames (ORFs). Electron microscopy showed that the viral genome is packaged as a filamentous particle with an average length of approximately 850 nm. Phylogenetic analysis and amino acid similarity analysis of the encoded ORFs revealed that this new virus could be classified in an intermediate position between the families *Benyviridae* and *Virgaviridae*. The GBCV 200-kDa replicase (ORF1) is more similar to benyvirus replicases than to virgavirus replicases, while its 17-kDa coat protein (CP, ORF2) is more closely related with virgavirus CPs than benyvirus CPs. ORF3 was predicted to produce a C-terminally extended protein from ORF2 via frameshifting. While ORF4 (45-kDa), ORF5 (44-kDa), and ORF6 (16-kDa) have no apparent sequence homology with other known viruses, ORF5 is predicted to encode a movement protein (MP) that is phylogenetically related to the furovirus MP and ORF6 was experimentally proven to encode a viral suppressor of RNA silencing. These unusual characteristics suggest that GBCV may represent an evolutionary link between the families *Benyviridae* and *Virgaviridae* and indicate the existence of a novel, unidentified virus group.

## Introduction

Since the first discovery of the *Tobacco mosaic virus* (TMV) in 1898, over 1470 species of plant viruses are now recognized by the International Committee for Taxonomy of Viruses (ICTV) [1]. However, these likely represent only a small fraction of plant virus biodiversity, because much of what has been described has been restricted to viruses that cause symptomatic diseases in crop plants. Recent studies have revealed an abundance of viruses even in apparently healthy wild plants, suggesting that plant viruses may be ubiquitous in wild plants [2–6]. This also suggests that there may be many evolutionary missing links that should be addressed to give an accurate picture of the nature of the plant virosphere and to increase our understanding of virus evolution and ecology [7].

Recent advances in next generation sequencing (NGS) technologies and bioinformatics are providing us a more in-depth picture of plant virus biodiversity [6, 8, 9]. This powerful approach allows the continuous identification of novel virus species, quantitative characterization of a mixed infection of different viruses, and meta-transcriptomic analysis of the diversity of variants of known viruses [8, 10, 11]. Recently, a large-scale meta-transcriptomic survey of invertebrate RNA viruses from over 220 invertebrate species sampled across nine anima phyla revealed 1445 RNA viruses that could fill major gaps in the RNA virus phylogeny [12]. Similar comprehensive approaches to understand plant virus biodiversity in cultivated and wild plants will enable us to re-define current classification schemes and illustrate a more detailed evolutionary history of plant viruses.

Plant viruses with single-stranded RNA genomes form a very diverse group with an enormous variation in genome structure and gene expression strategy. They could be classified into subgroups based on phylogenetic relationships determined by sequence homologies among the conserved virus genes, including those for the RNA-dependent RNA polymerase (RdRp), coat protein (CP), and movement protein (MP) [1]. Moreover, the comparison of the complete genome sequence and structure of RNA viruses has revealed an evolutionary phenomenon that involves gene module shuffling among diverse virus genomes [13, 14]. For example, diverse plant RNA viruses belonging to the families *Alphaflexiviridae*, *Betaflexiviridae*, *Benyviridae*, and *Virgaviridae* encode a similar element of three partially overlapping open reading frames (ORFs) called the triple gene block (TGB) that is involved in the cell-to-cell and long-distance movement of viruses [15]. This conservation of a specialized gene module may indicate the existence of unrevealed evolutionary links among these distantly related viruses.

In this study, we identified by Illumina RNA sequencing (RNA-Seq) an unusual filamentous plant virus, provisionally named goji berry chlorosis virus (GBCV), from goji berry plants (*Lycium chinense* Miller) showing virus-like symptoms of chlorosis. Its complete genome consists of a positive-sense single-stranded RNA of 10,100 ribonucleotides with a poly(A) tail at the 3’-end. Comparison of the genome organization and identities of the putative gene products of this new virus with those of previously described viruses show that GBCV could be classified in an intermediate position between the families *Benyviridae* and *Virgaviridae*, suggesting the existence of veiled evolutionary links between these two virus families.

## Materials and methods

### Ethics statement

Goji berry samples showing virus-like symptoms were collected from private fields under the permission of the owners of the fields. The field studies performed in this study did not involve endangered or protected species.

### Virus origin, electron microscopy, and RNA extraction

Leaf samples of goji berry showing virus-like symptoms were collected from commercial fields in Cheongyang City, Korea in June 2017. The collected samples were divided into two parts to examine whether they were infected with viruses. One part was processed for transmission electron microscopy (TEM) after negative staining with 2% uranyl acetate (pH 4.5). The other part was processed for RNA extraction using PureLink RNA Mini Kit (Invitrogen, Carlsbad, CA) according to the manufacturer’s instructions.

### Library construction and RNA sequencing

Total RNA extracted from the collected leaf samples were subjected to library construction using Illumina TruSeq RNA Sample Preparation Kit v2 (Illumina, Inc., USA) with no modifications to the standard protocol. RNA-Seq was performed using an Illumina HiSeq2000 sequencer (Illumina, Inc., USA). *De novo* assembly of the quality filtered RNA-Seq reads was performed using the Trinity pipeline and assembled contigs were analyzed by BLASTn and BLASTx searches against the viral reference genome database in GenBank [10, 16]. The entire RNA-Seq procedure was performed by Macrogen Inc. (Seoul, South Korea).

### Sequence analysis

Sequence identities were analyzed by comparing with sequences in GenBank by BLASTx. ORFs and conserved motifs were predicted using the ORF finder and the Conserved Domain Database (CDD), respectively, through the NCBI website (http://www.ncbi.nlm.nih.gov/). Hidden Markov Model analysis were performed using HMMER software (http://hmmer.org/). RNA structure prediction was performed by mfold software (http://unafold.rna.albany.edu/?q=mfold). Amino acid sequence identity between GBCV and the selected viruses in the families *Benyviridae* and *Virgaviridae* was analyzed by MegAlign software (Lasergene, DNAStar, Madison, WI).

### Phylogenetic analyses

The phylogenetic relationship of GBCV was analyzed by the maximum likelihood method implemented in the MEGA7 program using sequence alignments generated by the ClustalX program [17, 18]. Bootstrap values were calculated using 1000 random replications. The calculated trees were displayed using Tree Explorer implemented in the MEGA7 program. Representative species in the families *Benyviridae*, *Virgaviridae*, *Alphaflexiviridae*, and *Betaflexiviridae* were included in the phylogenetic analyses. Their GenBank accession numbers are as follows: apple stem pitting virus (ASPV; KY242757), broad bean necrosis virus (BBNV; NC_004423), burdock mottle virus (BdMoV, NC_021735), blueberry scorch virus (BlScV; AY941198), beet necrotic yellow vein virus (BNYVV; NC_003514), beet soil-borne mosaic virus (BSBMV; NC_003506), beet soil-borne virus (BSBV; NC_003520), beet virus Q (BVQ; NC_003510), cucumber green mottle mosaic virus (CGMMV; NC_001801), cherry necrotic rusty mottle virus (CNRMV; NC_002468), Colombian potato soil-borne virus (CPSbV; NC_029034), gentian ovary ring-spot virus (GORV; NC_024501), Indian citrus ringspot virus (ICRSV; NC_003093), Indian peanut clump virus (IPCV; NC_004729), lettuce virus X (LVX; NC_010832), peanut clump virus (PCV; NC_003672), pea early browning virus (PEBV; NC_002036), pepper ringspot virus (PepRSV; NC_003669), pepper mild mottle virus (PMMoV; NC_003630), potato mop-top virus (PMTV; KY275269), potato virus X (PVX; NC_011620), rice stripe necrosis virus (RSNV; EU099844), soil-borne wheat mosaic virus (SBWMV; NC_002041), shallot virus X (ShVX; NC_003795), tobacco mosaic virus (TMV; NC_001367), tobacco rattle virus (TRV; NC_003805), and tulip virus X (TVX; NC_004322).

### Transgene silencing suppression assay

The agroconstruct PZP-GFP expressing green fluorescence protein (GFP) mRNA *in planta* was described previously [19]. The GBCV ORF4, ORF5, and ORF6 were amplified by RT-PCR using appropriate primer sets (Primer information is available upon request) and inserted into the PZP vector utilizing *Stu*I and *Spe*I sites. The resulting constructs were referred to as PZP-GBCV-ORF4, PZP-GBCV-ORF5, and PZP-GBCV-ORF6, respectively. For the transgene silencing suppression assay [20], equal volumes of agrobacteria harboring PZP-GFP and either PZP-GBCV-ORF4, -ORF5, or -ORF6 were mixed and infiltrated into leaves of a *Nicotiana benthamiana* transgenic line expressing GFP (line 16c). At 3 dpi, the GFP fluorescence in the infiltrated leaves was examined using a hand-held long wave UV-light source (Blak-Ray B-100AP, Ultraviolet Products, USA).

## Results and discussion

### Nucleotide sequence and genome organization

**Fig 1:**
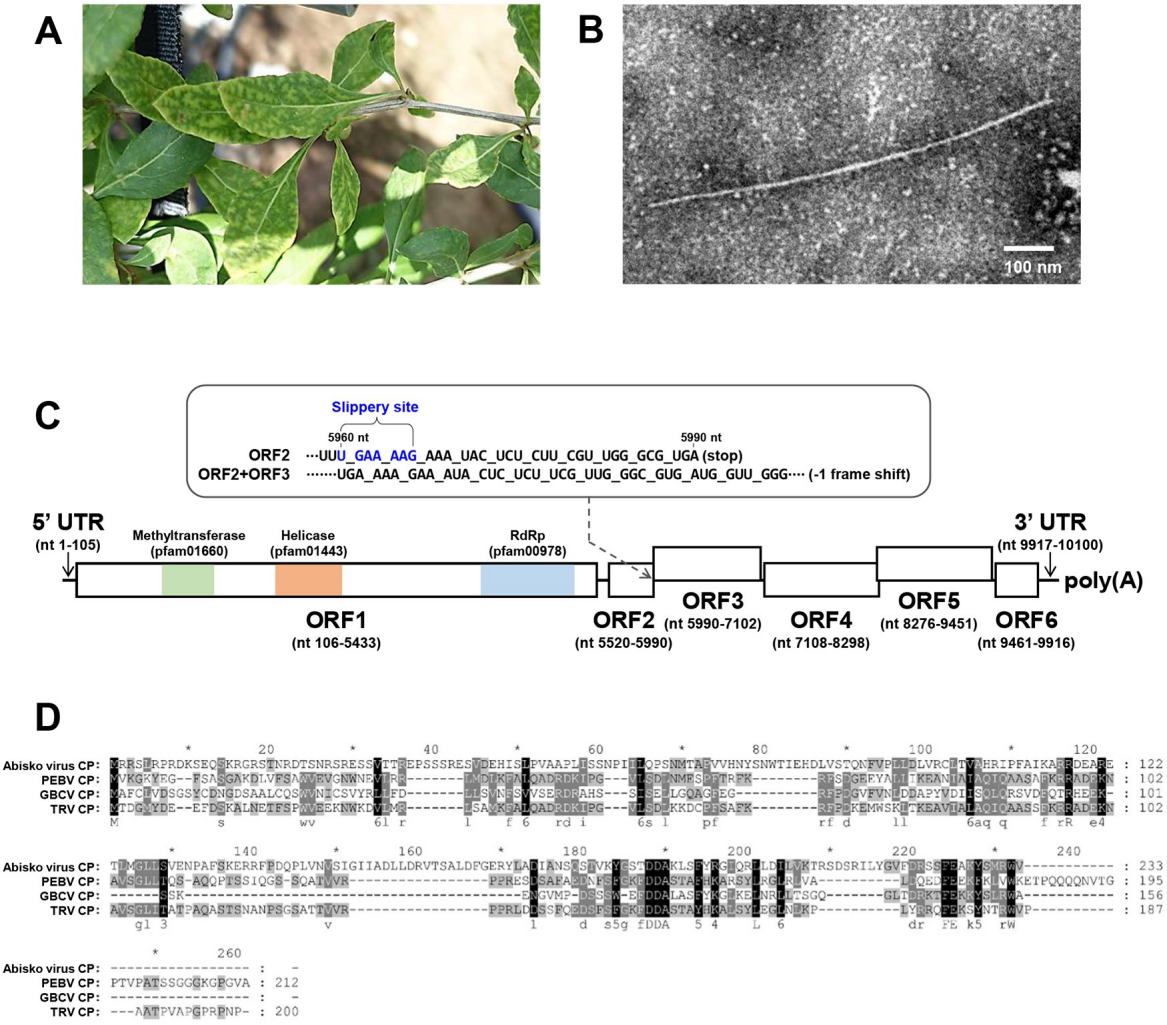
(A) Chlorosis symptoms on the leaves of a goji berry plant infected with goji berry chlorosis virus (GBCV). (B) Electron micrograph of negatively stained GBCV virion in a dip preparation of a symptomatic goji berry leaf. (C) The predicted genomic organization of GBCV. The GBCV genome contains six open reading frames (ORFs): ORF1, viral replicase protein (nt 106–5433, 200-kDa); ORF2, CP (nt 5520–5990, 17-kDa); ORF3, a putative CP-frameshifting protein (CPFS, nt 5990–7102); ORF4, an unknown protein (nt 7108–8298, 45-kDa); ORF5, a putative MP (nt 8276–9451, 44-kDa); ORF6, viral suppressor of RNA silencing (VSR, nt 9461–9916, 16-kDa). ORF3 is predicted to be expressed by -1 frame shifting at genomic nucleotide position 5960. ORF1 contains three conserved domains: viral methyltransferase domain (amino acid positions 286–470), viral helicase 1 domain (amino acid positions 675–902), and a RNA-dependent RNA polymerase (RdRp) core motif (amino acid positions 1377–1696). (D) Amino acid sequence alignment of the coat proteins (CPs) of GBCV, abisco virus (YP_009408587), pea early-browning virus (PEBV; CAA07067PEBV), and tobacco rattle virus (TRV; ABE27877). Highly conserved residues are highlighted.

Goji berry, which belongs to the *Solanaceae* family, is a commercially important crop and widely cultivated in Asia. In June 2017, during a survey conducted in commercial goji berry fields in Cheongyang City, Korea, virus-like symptoms of chlorosis were observed on the leaves of many goji berry plants (Fig 1A). The symptomatic leaves were collected and tested for the identification of the causal agent(s). Electron microscopic observation of negatively stained preparations from the collected leaf samples revealed the presence of flexuous filamentous virus-like particles with an average length of approximately 850 nm (Fig 1B). Although goji berry has been grown worldwide, viruses infecting goji berry are rather unknown. Thus, to identify the causal agent(s), the total RNA extracted from this sample was subjected to RNA-Seq as described previously [10, 16].

A total of 43,584,935 raw reads obtained after RNA-Seq were *de novo* assembled into contigs and analyzed against the viral genome reference database in GenBank. BLASTx search revealed one large contig (9976 nt) with homology to viruses. To confirm the RNA-Seq result, RT-PCR was performed to amplify the entire sequence of the contig from the total RNA subjected to RNA-Seq using the overlapping primer pairs designed based on the contig sequence obtained by RNA-seq (data not shown). The sequence of the amplified product was confirmed by *de novo* sequencing to be identical to that of the contig obtained by RNA-Seq. To obtain a full-length sequence, the terminal sequences of the contig were determined by the 5' and 3' RACE. The analysis of the 3’ end sequence showed that the contig contains a poly(A) tail at the 3’ end. The assembled full-length sequence of the contig comprised 10,100 nt, excluding the poly(A) tail. ORF prediction showed that the contig contains six ORFs flanked by a 5’ untranslated region (URT, 105 nt) and a 3’ UTR (184 nt) (Fig 1C). The ORFs were predicted to encode the following putative proteins: ORF1, viral replicase protein (nt 106–5433, 200-kDa); ORF2, CP (nt 5520–5990, 17-kDa); ORF3, a putative CP-frameshifting protein (CPFS, nt 5990–7102); ORF4, an unknown protein (nt 7108–8298, 45-kDa); ORF5, a putative MP (nt 8276–9451, 44-kDa); and ORF6, a viral suppressor of RNA silencing (VSR, nt 9461–9916, 16-kDa). The viral replicase protein encoded in ORF1 shares a significant, but low sequence identity with benyviruses [a maximum amino acid sequence similarity of 43% to the replicase protein (GenBank Accession No. ACA63029) of *Beet necrotic yellow vein virus* (BNYVV; genus *Benyvirus*; family *Benyviridae*)]. Therefore, we suggest that this contig is the genome sequence of a novel plant virus and the name goji berry chlorosis virus (GBCV) is proposed. The full-length genome sequence of GBCV was deposited in GenBank under the accession number MH791331.

ORF1 (replicase) of GBCV contains three conserved domains (Fig 1C): a viral methyltransferase domain (MTR, Accession No. pfam01660, amino acid positions 286–470, E-value 2.15e-06); a viral helicase 1 domain (HEL, pfam01443, amino acid positions 675–902, E-value 6.85e-25); and an RdRp core motif (pfam00978, amino acid positions 1377–1696, E-value 9.20e-10). The MTR domain is conserved in a wide range of single-stranded RNA viruses, including tobra-, tobamo-, bromo-, and closteroviruses [21]. The HEL domain contains the conserved motifs GKS and DE [13], which were identified from amino acid positions 682–684 and 731–732, respectively. The RdRp motif contains the conserved critical motif S/TG(X_3_)T(X_3_)NS/T(X_22_)GDD (where X is any residue) at amino acid positions 1584–1619 [22]. Although the GBCV replicase shows a marginal similarity to benyvirus replicases, no papain-like proteinase domain, which is conserved in benyvirus replicases and required to produce mature replicases by proteolytic cleavage [23], was predicted in the GBCV replicase.

ORF2 is predicted to encode a 17-kDa viral CP based on a BLASTx search showing that it has marginal amino acid similarity with the abisko virus (unclassified insect RNA virus) CP (YP_009408587, 29% identity with 87% coverage, E-value 1e-07), with the tobacco rattle virus (TRV; genus *Tobravirus*; family *Virgaviridae*) CP (ABE27877, 26% identity with 94% coverage, E-value 0.009), and with the pea early-browning virus (PEBV; genus *Tobravirus*; family *Virgaviridae*) CP (CAA07067, 25% identity with 95% coverage, E-value 1.6). Amino acid sequence alignment revealed the GBCV CP contains some conserved motifs, such as DDA and FE, which were identified from amino acid positions 77–79 and 147–148, respectively (Fig 1D).

**Fig 2:**
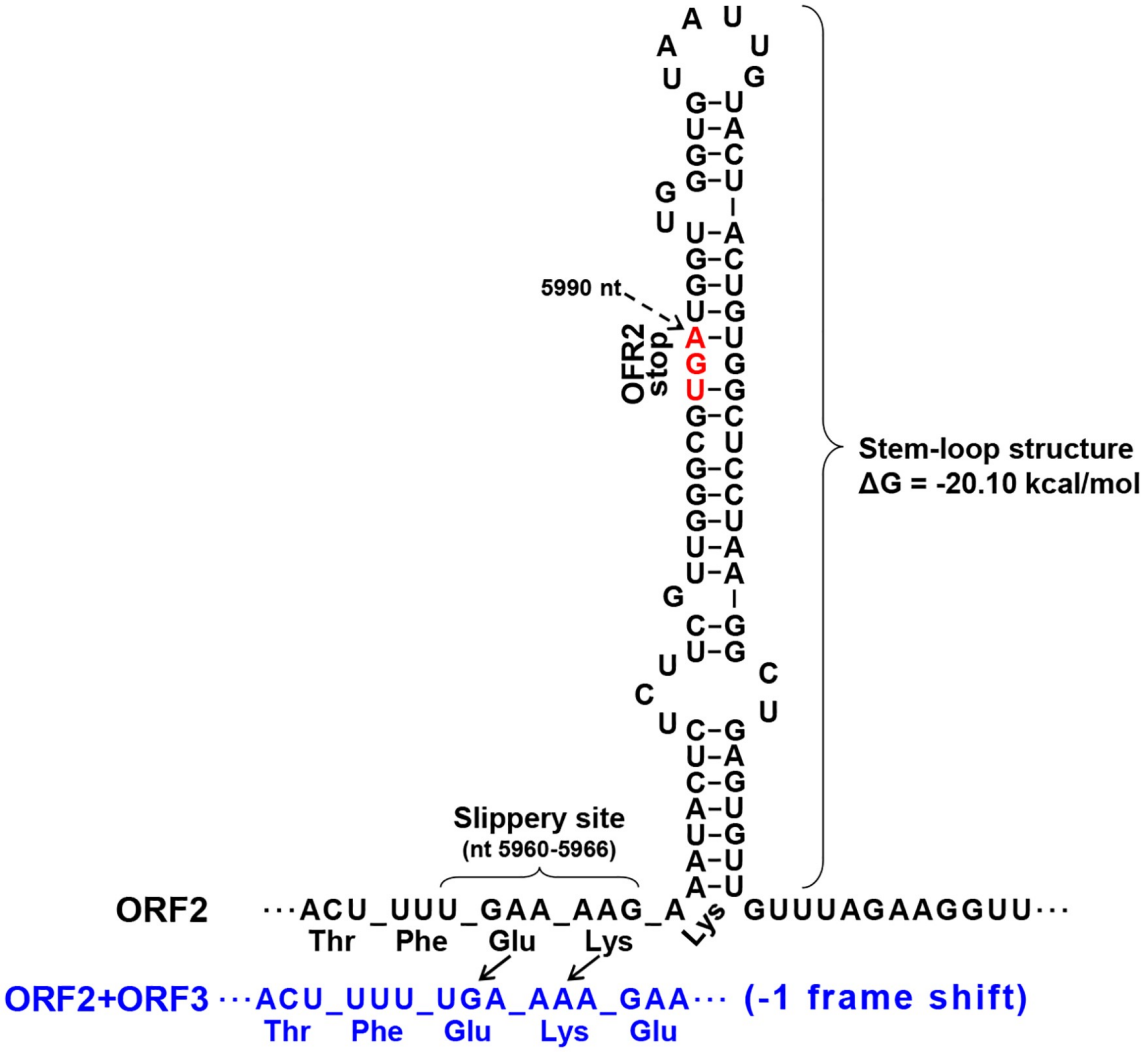
The predicted essential elements for -1 ribosomal frameshifting at the ORF2/ORF3 overlap region. The slippery site is the heptamer UGAAAAG (nt 5960–5966). The stem-loop structure just downstream to the slippery site is shown. The ORF2+ORF3 fusion frame generated by -1 ribosomal frameshifting is shown in the lower row (blue). The two tRNAs (Glu and Lys) bound on the ribosomes to the GAA AAG on the slippery site slip back 1 base each to bind to UGA AAA.

ORF3 was detected in nt 5990–7102 and its N-terminus overlapped ORF2 in frame -1 (Fig 1C). ORF3 has 27% identity (39% coverage, E-value 2e-06) with the TRV CP (AAC02063). Similar overlapping between two ORFs have been found in various RNA viruses that employ -1 ribosomal frameshifting to control when the translation of an ORF terminates at a stop codon or continues in the new reading frame to produce another C-terminally extended protein [24, 25]. Those RNA viruses contain cis-acting frameshift signals typically composed of a slippery sequence and a stem-loop structure element positioned just downstream of the slippery site for efficient -1 ribosomal frameshifting [24–26]. Indeed, computational analysis predicted putative slippery heptameric sequences (UGAAAAG; nt 5960–5966) and a stem-loop structure near the ORF2/ORF3 overlap region (Fig 2). The predicted slippery site and stem-loop structure and the distance between the two elements resemble those of other RNA viruses employing a ribosomal frameshifting mechanism [24–26]. However, we cannot exclude another possibility that ORF3 may be translated by a ribosomal leaky scanning mechanism as found in peanut clump virus (PCV; genus *Pecluvirus*; family *Virgaviridae*), which has a similar genetic organization with GBCV [27]. Further experimental investigations are required to elucidate the real mechanism.

ORF4 and ORF5 were calculated to encode 45-kDa and 44-kDa proteins, respectively (Fig 1C). BLASTn and BLASTx analyses showed that ORF4 and ORF5 have no apparent sequence homology with known viruses. The NCBI CDD analysis identified three possible domain hits in ORF4, including the SMC_N superfamily (Accession No. TIGR02169, amino acid positions 124–378, E-value 1.55e-05), Spc7 superfamily (Accession No. smart00787, amino acid positions 220–371, E-value 5.05e-04), and Herpes_UL36 superfamily domains (Accession No. PHA03246, amino acid positions 213–300, E-value 5.78e-03), but no putative conserved domains were detected in ORF5. The SMC_N superfamily domain is found at the N terminus of SMC (structural maintenance of chromosomes) proteins that bind DNA and act in organizing and segregating chromosomes [28]. The Spc7 superfamily domain is found in cell division proteins that are required for kinetochore-spindle association [29]. The herpesvirus UL36 protein is a component of the virion tegument [30]. In addition, another protein domain analysis using HMMER software predicted two coiled-coil motifs, located at amino acid positions 139–166 and 224–244 in ORF4. A coiled-coil motif is a well-known protein structural motif important for binding to DNA and RNA [31]. Thus, it is likely that the ORF4 protein has viral RNA binding activity required for viral replication, encapsidation, or movement.

**Fig 3:**
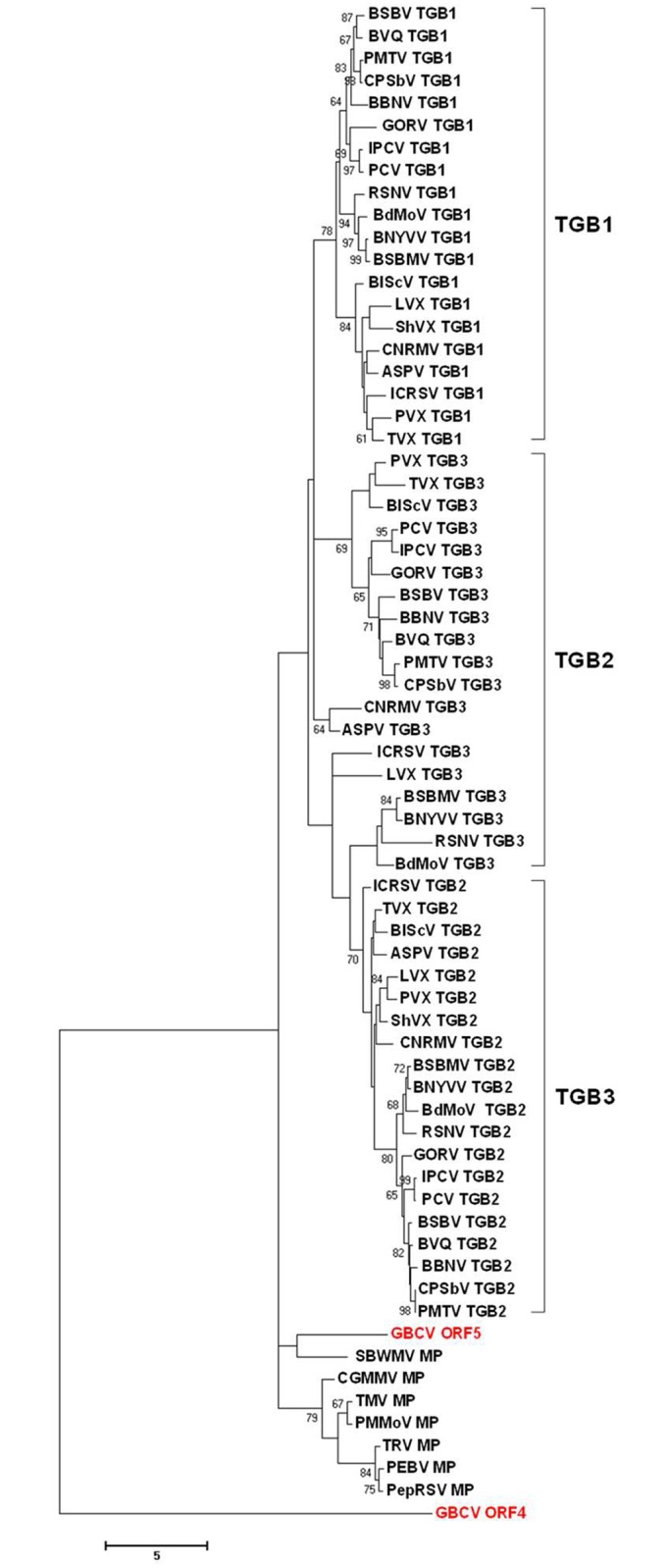
Phylogenetic relationships of the GBCV ORF4 and ORF5 products to the movement proteins (MPs) of representative members in the families *Benyviridae*, *Virgaviridae*, *Alphaflexiviridae*, and *Betaflexiviridae*. Phylogenetic trees were constructed using the Maximum likelihood method with the MEGA7 program based on an alignment of the MP amino acid sequences. The numbers on the branches indicate bootstrap percentages (only values >60% are shown) based on 1000 replications. The full names of the viruses and accession numbers used in the phylogenetic analysis are described in the Materials and Methods section.

Plant viruses encode MPs for viral cell-to-cell and long-distance trafficking [32]. As described below, ORF6 (16-kDa) was found to encode a VSR. Thus, we expected that either ORF4 or ORF5 might encode an MP. Recognized MPs can be classified largely into four superfamilies: the “30K” superfamily, related to the TMV MP; the TGB proteins of potexviruses and related viruses; the tymoviral MPs; and a series of small proteins, less than 10 kDa, encoded by carmo-like viruses and some geminiviruses [15, 32, 33]. Thus, we sought to examine if either ORF4 or ORF5 has phylogenetic relationships with other known viral MPs. The phylogenetic tree was reconstructed by the maximum likelihood method using the MP amino acid sequences of various viruses that belong to the families *Benyviridae*, *Virgaviridae*, *Alphaflexiviridae*, and *Betaflexiviridae* (Fig 3). The tree showed that ORF5 has a close relationship with the MP of soil-borne wheat mosaic virus (SBWMV; genus *Furovirus*; family *Virgaviridae*), whereas ORF4 has no phylogenetic relationship with other viral MPs, suggesting that ORF5 may encode a viral MP. Further experimental investigations are required for functional analyses of the ORF5 translation product.

**Fig 4:**
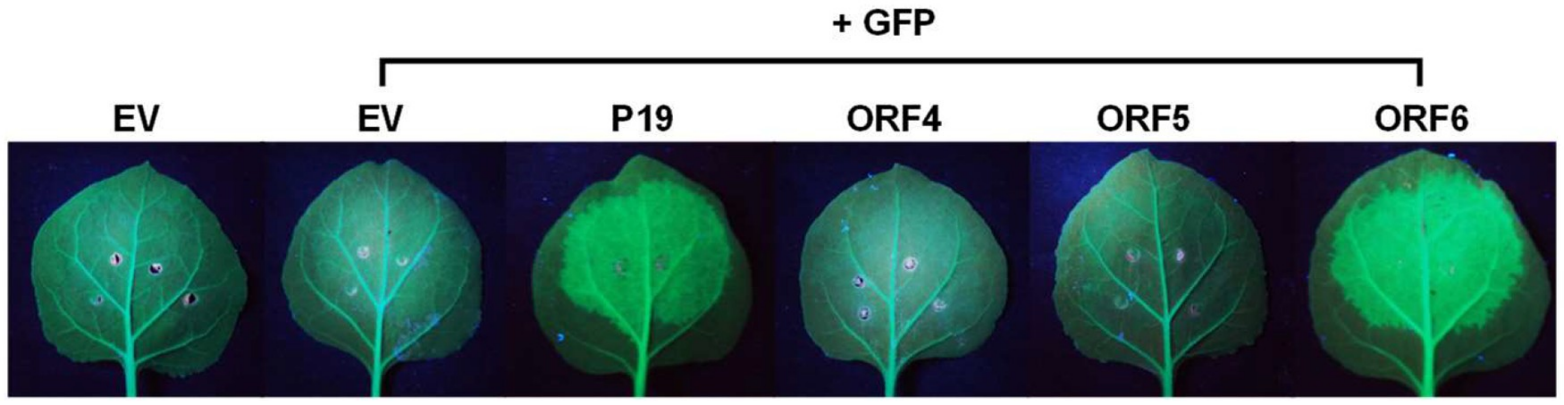
Evaluation of silencing suppression activities of the GBCV ORF4, ORF5, and ORF6. Leaves of 16c GFP transgenic plants were infiltrated with agrobacteria expressing GFP together with agrobacteria expressing TBSV P19, GBCV ORF4, ORF5, or ORF6. At 3 dpi, the green fluorescence images of the infiltrated leaves were taken under UV light. EV means agrobacteria carrying an empty vector.

RNA silencing is an important part of innate immunity against viruses in plants [34, 35]. To counter this host defense, plant viruses encode the viral proteins known as VSRs that have the ability to inhibit RNA silencing [36, 37]. To examine if GBCV encodes a VSR, ORF4, ORF5 and ORF6 were tested by a GFP transgene silencing suppression assay [20, 38, 39]. The agrobacteria carrying the PZP-GFP binary vector was co-infiltrated with agrobacteria expressing either ORF4, ORF5, or ORF6 into leaves of a *N*. *benthamiana* transgenic line expressing GFP (line 16c). The tomato bushy stunt virus (TBSV) P19, which is a well-characterized viral silencing suppressor, was used as a positive control. At 3 dpi, GFP expression was observed under UV light. Suppression of GFP silencing was observed in the leaves infiltrated with agrobacteria expressing ORF6, but not in plants infiltrated with agrobacteria expressing either ORF4 or ORF5 (Fig 4), indicating that ORF6 has strong RNA silencing suppression activity.

### Phylogenetic analysis

The taxonomic position of GBCV was investigated by phylogenetic analyses of the complete genomic nucleotide and amino acid sequences of encoded proteins of the virus. The phylogenetic trees were generated by including various virus species in the family *Benyviridae* and other related virus species that belong to the families *Virgaviridae*, *Alphaflexiviridae*, and *Betaflexiviridae*. As described above, the GBCV replicase, CP, and MP were found to be most closely related with the BNYVV replicase, TRV CP, and SBWMV MP, respectively. However, BNYVV, TRV, and SBWMV are multipartite viruses and their rod-shape virions are smaller than 400 nm in length [23, 40], whereas GBCV has a non-segmented genome and the size of its filamentous particles is approximately 850 nm. On the other hand, some viruses in the families *Alphaflexiviridae* and *Betaflexiviridae* have similar genome organizations and virion sizes as GBCV [41, 42], although they have no significant sequence similarities with GBCV.

**Fig 5:**
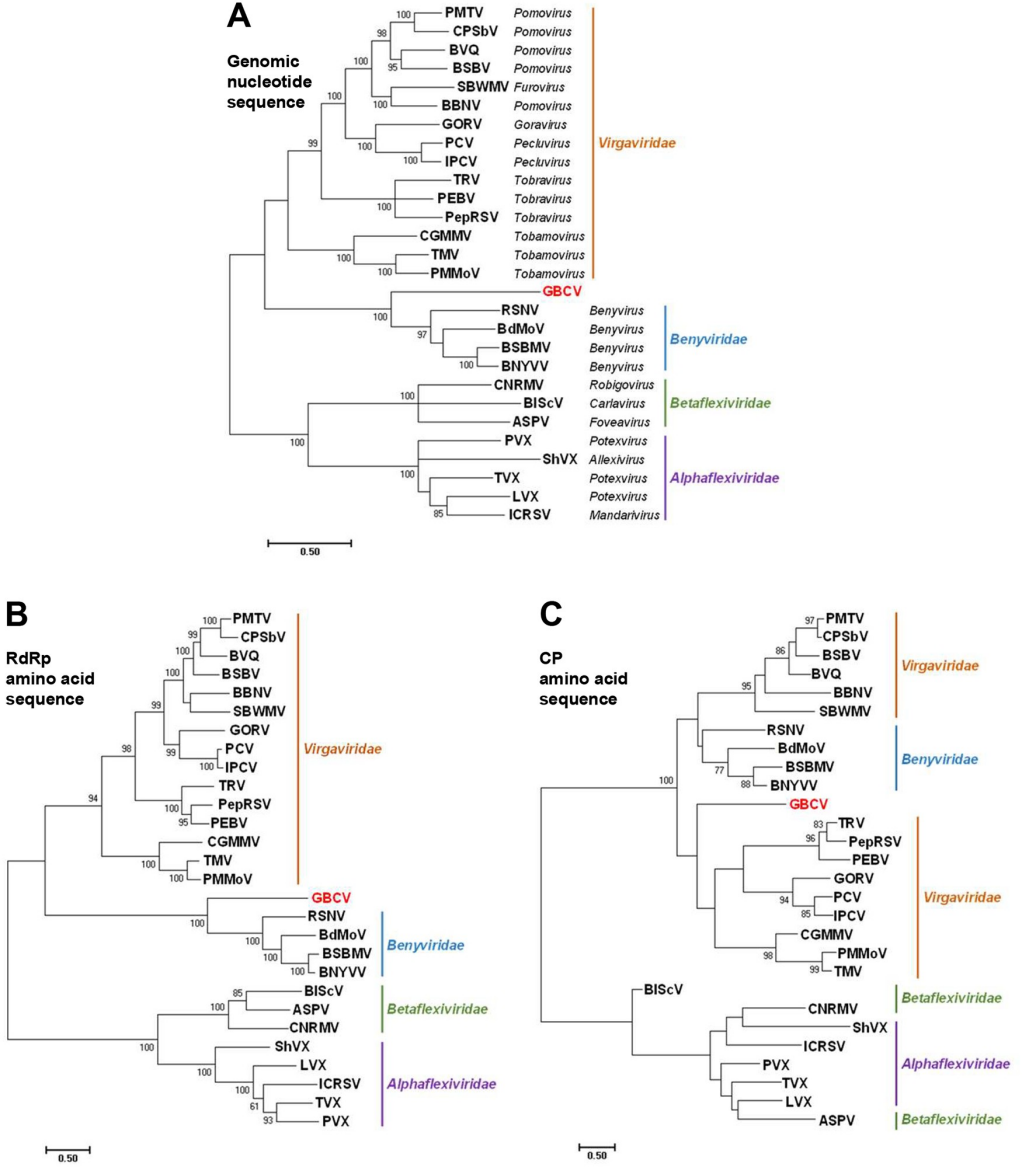
Evolutionary relationships of GBCV to representative species in the families *Benyviridae*, *Virgaviridae*, *Alphaflexiviridae*, and *Betaflexiviridae* phylogenetic trees were constructed using the maximum likelihood method with the MEGA7 program based on an alignment of the genomic nucleotide sequences (A), replicase (RdRp) amino acid sequences (B), or coat protein (CP) amino acid sequences (C) of selected members of the families *Benyviridae*, *Virgaviridae*, *Alphaflexiviridae*, and *Betaflexiviridae*. The numbers on the branches indicate bootstrap percentages (only values >60% are shown) based on 1000 replications. The full names of the viruses and accession numbers are described in the Materials and Methods section.

As the viruses in the families *Benyviridae* and *Virgaviridae* have segmented genomes [23, 40], their RNA1 nucleotide sequences, which encode viral replicases, were used for the phylogenetic analysis of the genomic nucleotide sequence of GBCV. The tree generated using the viral genomic nucleotide sequences shows GBCV forming a branch more closely associated with benyviruses (Fig 5A). A similar phylogenetic tree was also obtained when the replicase amino acid sequences of the viruses were analyzed (Fig 5B). However, the tree constructed using the CP amino acids shows that GBCV is slightly closer to tobraviruses than benyviruses (Fig 5C). In addition, as shown above, phylogenetic analysis showed GBCV is likely to encode an MP in ORF5 that is related with the SBWMV MP (Fig 3), while benyviruses encode the TGB proteins for virus movement [23]. Pairwise comparisons of the amino acid sequences of the replicase and CP of GBCV against other viruses further supports the view that GBCV cannot be categorized into the current taxonomic classification: The GBCV replicase shared the highest amino acid identity (25.5–28.9%) with those of the members in the genus *Benyvirus*, while the GBCV CP had the highest amino acid identity with those of tobraviruses (23.7–27%) (Table 1). This phylogenetic incompatibility of GBCV suggests that GBCV may represent an evolutionary link between the families *Benyviridae* and *Virgaviridae*. In addition, significant differences in virion morphology and genome organization between GBCV and the viruses belonging to the families *Benyviridae* and *Virgaviridae* indicate the existence of a novel unidentified virus group that includes GBCV.

**Table 1:** Amino acid sequence identities (%) of the genes encoded by goji berry chlorosis virus compared with those of selected viruses in the families *Benyviridae* and *Virgaviridae*.

Family	*Benyviridae*				*Virgaviridae*									
Genus	*Benyvirus*				*Tobravirus*			*Tobamovirus*			*Furovirus*	*Pomovirus*		
Species	BNYVV	BSBMV	BurMoV	RSNV	TRV	PepRSV	PEBV	TMV	PMMoV	CGMMV	SBWMV	BBNV	BSBV	BVQ
Replicase	25.5	26.5	28.9	26.9	8.3	8.8	7.9	10.3	10.9	9.3	7.8	7.7	6.0	5.9
CP	14.1	15.4	16.7	11.5	27.0	23.7	23.7	19.9	17.9	21.8	14.1	11.5	19.2	19.9

Recent advances in high-throughput virus detection technologies have allowed an explosion of novel virus species to be identified and are providing more detailed clues to redefine the virosphere [6, 8, 9]. A large-scale metagenomic analysis of virus diversity in vertebrates and invertebrates showed that many newly identified animal viruses belong to the virus families that were previously only known to infect plants, fungi, and protists [12, 43], indicating that there exists big unknowns in our knowledge of virus biodiversity. In wild plants, until recently, there was limited interest in virus biodiversity, but accumulating evidence shows that symptomless virus infection of wild plants is common in nature [2–6]. When considered as a long-term host-virus co-evolutionary process, symptomless adaptation of a virus to a host is beneficial for survival and many unidentified plant viruses might be asymptomatic. A recent study using a high-throughput RNA-seq approach has identified an unusual plant virus, designated donkey orchid symptomless virus (DOSV), from asymptomatic wild plants of common donkey orchid [5]. Interestingly, DOSV showed the considerable genetic deviation from known plant viruses: The DOSV replicase and CP have homology to those of alphaflexiviruses, while the MP resembles homologues found in tombus-like viruses. DOSV now classified into the genus *Platypuvirus* in the family *Alphaflexiviridae* might represent an evolutionary link between alphaflexiviruses and tombus-like viruses. Similarly, GBCV contains a chimeric genetic composition between benyviruses and virgaviruses (Fig 5 and Table 1). Such modular genome evolution indicates that the genome organization of GBCV is highly flexible, allowing changes in gene order and genome segmentation [13, 14]. Indeed, benyviruses, which are most closely related to GBCV, have segmented genomes and encode the TGB proteins for virus movement. The TGB unit may represent the modular genome evolution in RNA viruses. This distinctive viral MP might have been spread in largely distant viruses by exchanging the functional unit among viruses. Although it is unclear yet if GBCV should be placed at a more ancestral position than benyviruses in virus evolutionary history, the molecular genetic characteristics of GBCV support the modular theory of virus evolution previously suggested for bacteriophages [44].

## Conclusions

We describe the discovery of a previously unidentified plant RNA virus, provisionally named goji berry chlorosis virus (GBCV), isolated from goji berry plants showing chlorosis symptoms. Based on determination of the complete genome sequence of GBCV, its genome organization, putative proteome characteristics, and taxonomic position were analyzed. The principal biological significance of the discovery of GBCV is in the intermediate position that this virus occupies between two different virus families, the *Benyviridae* and *Virgaviridae*. Its replicase has more homology to those of benyviruses, while its CP is more closely related to those of virgaviruses. Nevertheless, the genome segmentation, virion shape, and some genetic compositions of GBCV are quite different from those of the members of either *Benyviridae* and *Virgaviridae*. These unusual characteristics of GBCV make it difficult to classify the virus according to the current virus taxa. The construction of an infectious cDNA clone of GBCV will be helpful for further biological and molecular characterization of this unusual plant RNA virus.

## Supporting information

S1 File Complete genome sequence of goji berry chlorosis virus. (TXT)
